# Longitudinal changes in magnetic resonance imaging biomarkers of the gluteal muscle groups and functional ability in Duchenne muscular dystrophy: a 12-month cohort study

**DOI:** 10.1007/s00247-023-05791-7

**Published:** 2023-10-27

**Authors:** Yu Song, Ke Xu, Hua-yan Xu, Ying-kun Guo, Rong Xu, Hang Fu, Wei-feng Yuan, Zi-qi Zhou, Ting Xu, Xi-jian Chen, Yi-lei Wang, Chuan Fu, Hui Zhou, Xiao-tang Cai, Xue-sheng Li

**Affiliations:** 1 Department of Radiology, Key Laboratory of Obstetric and Gynecologic and Pediatric Diseases and Birth Defects of Ministry of Education, West China Second University Hospital, Sichuan University, Chengdu, 610041 China; 2 Department of Rehabilitation Medicine, Key Laboratory of Obstetric and Gynecologic and Pediatric Diseases and Birth Defects of Ministry of Education, West China Second University Hospital, Sichuan University, Chengdu, 610041 China

**Keywords:** Duchenne muscular dystrophy, Gluteal muscle groups, Longitudinal relaxation time, Fat fraction, North star ambulatory assessment, Timed functional tests

## Abstract

**Background:**

Quantitative magnetic resonance imaging (MRI) is considered an objective biomarker of Duchenne muscular dystrophy (DMD), but the longitudinal progression of MRI biomarkers in gluteal muscle groups and their predictive value for future motor function have not been described.

**Objective:**

To explore MRI biomarkers of the gluteal muscle groups as predictors of motor function decline in DMD by characterizing the progression over 12 months.

**Materials and methods:**

A total of 112 participants with DMD were enrolled and underwent MRI examination of the gluteal muscles to determine fat fraction and longitudinal relaxation time (T1). Investigations were based on gluteal muscle groups including flexors, extensors, adductors, and abductors. The North Star Ambulatory Assessment and timed functional tests were performed. All participants returned for follow-up at an average of 12 months and were divided into two subgroups (functional stability/decline groups) based on changes in timed functional tests. Univariable and multivariable logistic regression methods were used to explore the risk factors associated with future motor function decline.

**Results:**

For the functional decline group, all T1 values decreased, while fat fraction values increased significantly over 12 months (*P*<0.05). For the functional stability group, only the fat fraction of the flexors and abductors increased significantly over 12 months (*P*<0.05). The baseline T1 value was positively correlated with North Star Ambulatory Assessment and negatively correlated with timed functional tests at the 12-month follow-up (*P*<0.001), while the baseline fat fraction value was negatively correlated with North Star Ambulatory Assessment and positively correlated with timed functional tests at the 12-month follow-up (*P*<0.001). Multivariate regression showed that increased fat fraction of the abductors was associated with future motor function decline (model 1: odds ratio [OR]=1.104, 95% confidence interval [CI]: 1.026~1.187, *P*=0.008; model 2: OR=1.085, 95% CI: 1.013~1.161, *P*=0.019), with an area under the curve of 0.874.

**Conclusion:**

Fat fraction of the abductors is a powerful predictor of future motor functional decline in DMD patients at 12 months, underscoring the importance of focusing early on this parameter in patients with DMD.

## Introduction

Duchenne muscular dystrophy (DMD) is an X-linked severe muscle degenerative disease caused by mutations in the dystrophin gene [1]. Dystrophin maintains the integrity of muscle fibers and protects the sarcolemma from contraction-induced injury [2, 3]. Thus, dystrophin deficiency damages the stability and function of muscle fibers, leading to progressive muscle atrophy and loss of muscle fibers, which are eventually replaced by fat and fibrous tissue [4, 5].

Duchenne muscular dystrophy involves multiple systems, generally including skeletal muscle and the cardiorespiratory system [6–8]. Gait abnormality is usually the first symptom of DMD patients, and skeletal muscle degeneration leads to skeletal deformity and respiratory insufficiency, eventually culminating in death from respiratory and cardiac failure in the third decade of life [9, 10]. Therefore, it is particularly important to evaluate and predict skeletal muscle function in the early stage of DMD patients, which is crucial for the prevention of disease progression. A large number of studies have confirmed that quantitative MRI measurement is an objective biomarker of DMD [11–13]. Currently, quantitative MRI methods mainly include T1 mapping, T2 mapping, Dixon, diffusion tensor imaging (DTI), and magnetic resonance spectroscopy (MRS), among which functional MRI such as DTI and MRS can provide information about skeletal muscle substructure and metabolism [14]. Longitudinal relaxation time (T1) and fat fraction values obtained by multimodal quantitative MRI can reflect the pathological changes in muscle. The T1 value is mainly influenced by fibrosis, inflammation, and fat infiltration [15, 16], and the fat fraction value is mainly influenced by fat infiltration [17]. However, previous studies have mostly focused on individual muscles in DMD. Our team has previously demonstrated the potential of MRI biomarkers of the gluteal muscle group to characterize DMD motor dysfunction [18]. However, at present, there are no quantitative data on the longitudinal progression of fat infiltration, atrophy, or edema in gluteal muscle groups of DMD patients, and there is also a lack of highly sensitive and specific MR biomarkers based on gluteal muscle groups to describe disease progression in DMD. In addition, the contribution and predictive value of specific gluteal muscle groups to future clinical motor function have not been described.

Therefore, the purpose of this study is to describe the longitudinal progression of MR biomarkers of gluteal muscle groups and to correlate baseline MR biomarkers of gluteal muscle groups with future clinical motor function to ascertain the potential of different MR biomarkers to predict future motor functional decline.

## Materials and methods

### Study design and participants

From March 2020 to February 2022, 124 boys with DMD diagnosed by genetic testing and/or skeletal muscle pathology were included. After excluding 12 patients (five with poor image quality and seven did not cooperate sufficiently to complete functional testing), 112 boys with DMD were enrolled for baseline (mean age 9.1±1.7 years, range 4–14 years) and 12-month follow-up (mean age 10.20±1.70 years, range 5–15 years) measurement data. At the baseline visit, participants underwent MRI examination of the gluteal muscles, followed by clinical assessment of motor function. Participants returned at an average of 12.3±2.3 months for follow-up MR and functional data collection. This prospective study was reviewed and approved by the Institutional Review Board (clinical trial registration number: ChiCTR1800018340). Prior informed consent was obtained from the subjects’ guardians.

### Magnetic resonance imaging acquisition/analysis

Imaging was performed with a 3-tesla MR scanner (Magnetom Skyra, Siemens Healthineers, Erlangen, Germany) equipped with an 18-channel receiver coil. All patients underwent MRI scanning from the iliac crest to the mid-thigh. MRI protocols included T1 mapping and Dixon sequences. Sequence parameters were set as follows: T1 mapping was performed using a modified Look-Locker inversion recovery (MOLLI) sequence (echo time (TE)=1.1 ms, repetition time (TR)=2.7 ms, flip angle (FA)=35°, slice thickness=6 mm, matrix=400×340, acquisition time (T_acp_)=54 s). Quantitative water/fat imaging was performed by T2-weighted Dixon sequencing with fast spin echo (TE=55 ms, TR=3660 ms, FA=150°, slice thickness=6 mm, matrix=360×360, T_acp_=2 min 34 s).

### Functional outcomes

After the MR examinations were complete, participants performed North Star Ambulatory Assessment and timed functional tests, including the 10-m run/walk, 4-stair climb, 4-stair descend, and Gowers maneuver tests. The Gowers maneuver is the time required for patients to rise from a sitting position on the floor to standing [19, 20]. All subjects were divided into two subgroups (functional stability group and functional decline group) based on changes in timed functional tests over 12 months. The functional stability group was defined as a change between −0.5 s and 0.5 s in timed functional tests, and the functional decline group was defined as a change >0.5 s in more than half of the timed functional tests [11, 21].

### Imaging assessment

**Fig. 1:**
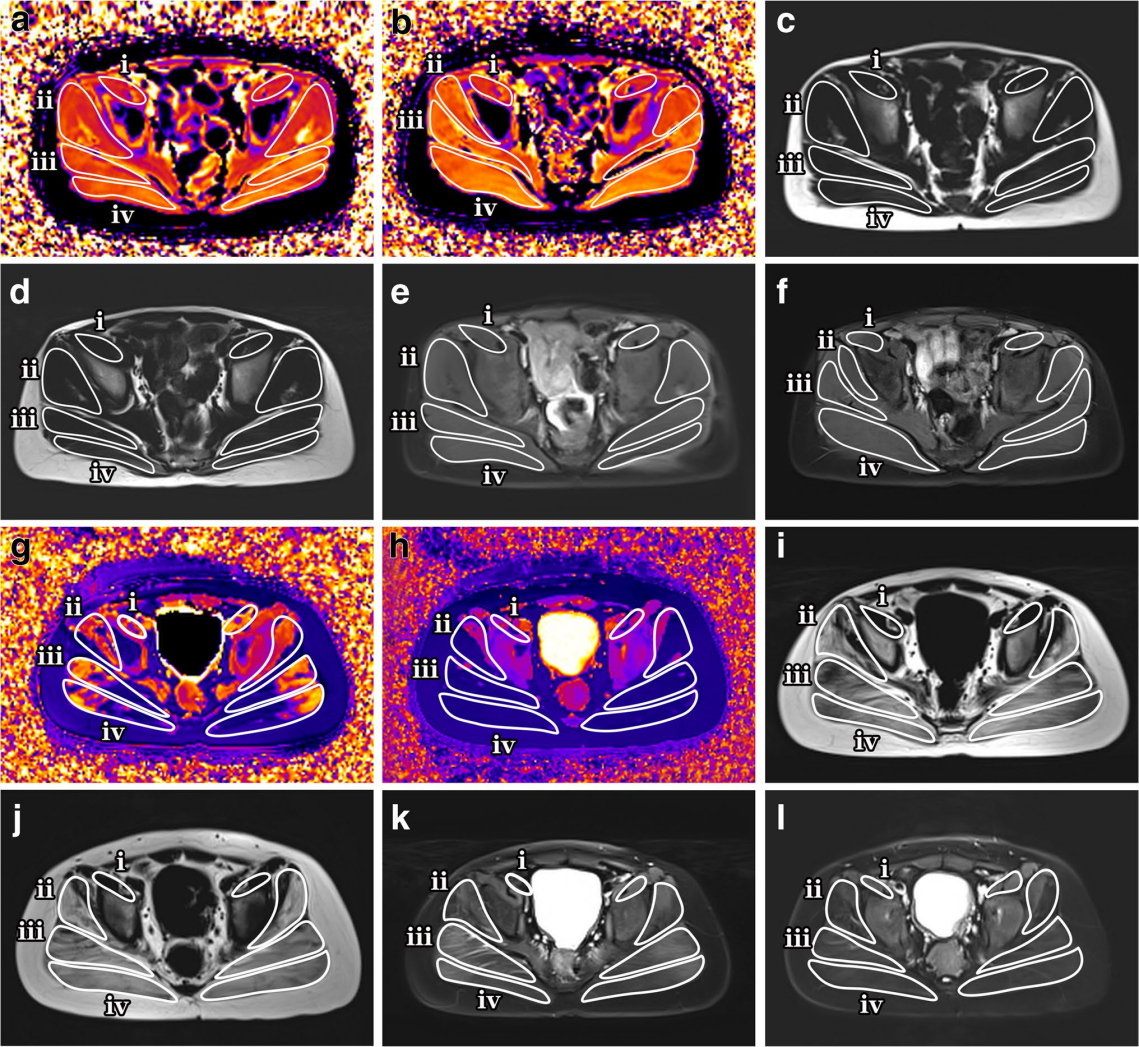
Axial magnetic resonance imaging of the gluteal muscles at ages 7 years and 8 months (baseline) and 8 years and 7 months (12-month follow-up) in a boy with Duchenne muscular dystrophy in the functional stability group (**a**–**f**) and the gluteal muscles at ages 9 years and 5 months (baseline) and 10 years and 6 months (12-month follow-up) in a boy with Duchenne muscular dystrophy in the functional decline group (**g**–**l**). Sequences shown are T1 maps at baseline (**a**, **g**) and 12-month follow-up (**b**, **h**), Dixon (F) at baseline (**c**, **i**) and 12-month follow-up (**d**, **j**) and Dixon (W) at baseline (**e**, **k**) and 12-month follow-up (**f**, **l**). *i* iliacus, *ii* gluteus minimus, *iii* gluteus medius, *iv* gluteus maximus

Data measurement was performed by two experienced radiologists (Y.S., a pediatric radiologist with 6 years of experience, and T.X., a pediatric radiologist with 6 years of experience) independently using a Siemens MR Postprocessing workstation (Syngo.Via, Erlangen, Germany). Investigations were all based on muscle groups covering the flexors (iliacus, rectus femoris, and sartorius), extensors (gluteus maximus, biceps femoris, semitendinosus, and semimembranosus), adductors (adductor magnus, adductor longus, adductor brevis, pubis, and gracilis), and abductors (gluteus medius, gluteus minimus, tensor fascia). Slices that contained the largest area of visible muscle with good differentiation of muscle groups were chosen for placing the region of interest (ROI), including four major cross-section levels: (a) at the sciatic foramen (Fig. 1); (b) at the greater trochanter-ischial tuberosity; (c) at the proximal femoral diaphysis; and (d) at the mid-point of the femoral diaphysis. The T1 maps were color coded pixel by pixel with colors corresponding to a range of T1 values. Placement of the ROI of each muscle on the T1 map allowed calculation of the mean T1 value. Fat fraction values were calculated as signal intensity (SI) fat/(SI _fat_+SI _water_)×100% from the reconstructed fat and water images [22]. The size of the ROI was determined by using the individual muscle size on the axial images.

### Statistical analysis

Statistical analysis was performed using Statistical Product and Service Solutions software (SPSS, version 26.0, IBM Crop, Armonk, NY) and MedCalc software (MedCalc Software Ltd, Ostend, Belgium). Values are presented as the mean ± standard deviation (SD). Comparison of continuous variables was performed using the nonparametric Mann–Whitney *U* rank sum test. We used univariate and multivariable logistic regression methods to explore the risk factors associated with decreased motor function in DMD. Receiver operator characteristic (ROC) curve analysis was performed to investigate the diagnostic value of the fat fraction of abductors in identifying motor function, and the area under the curve (AUC) was calculated. Consequently, Youden’s index was used to calculate the optimal cutoff value of DMD motor function decline. Spearman correlation coefficients were used to evaluate the correlation between baseline MR biomarkers and 12-month follow-up functional abilities, and a heatmap visualization was conducted to clarify the relationships.

## Results

### Participant characteristics

**Fig. 2:**
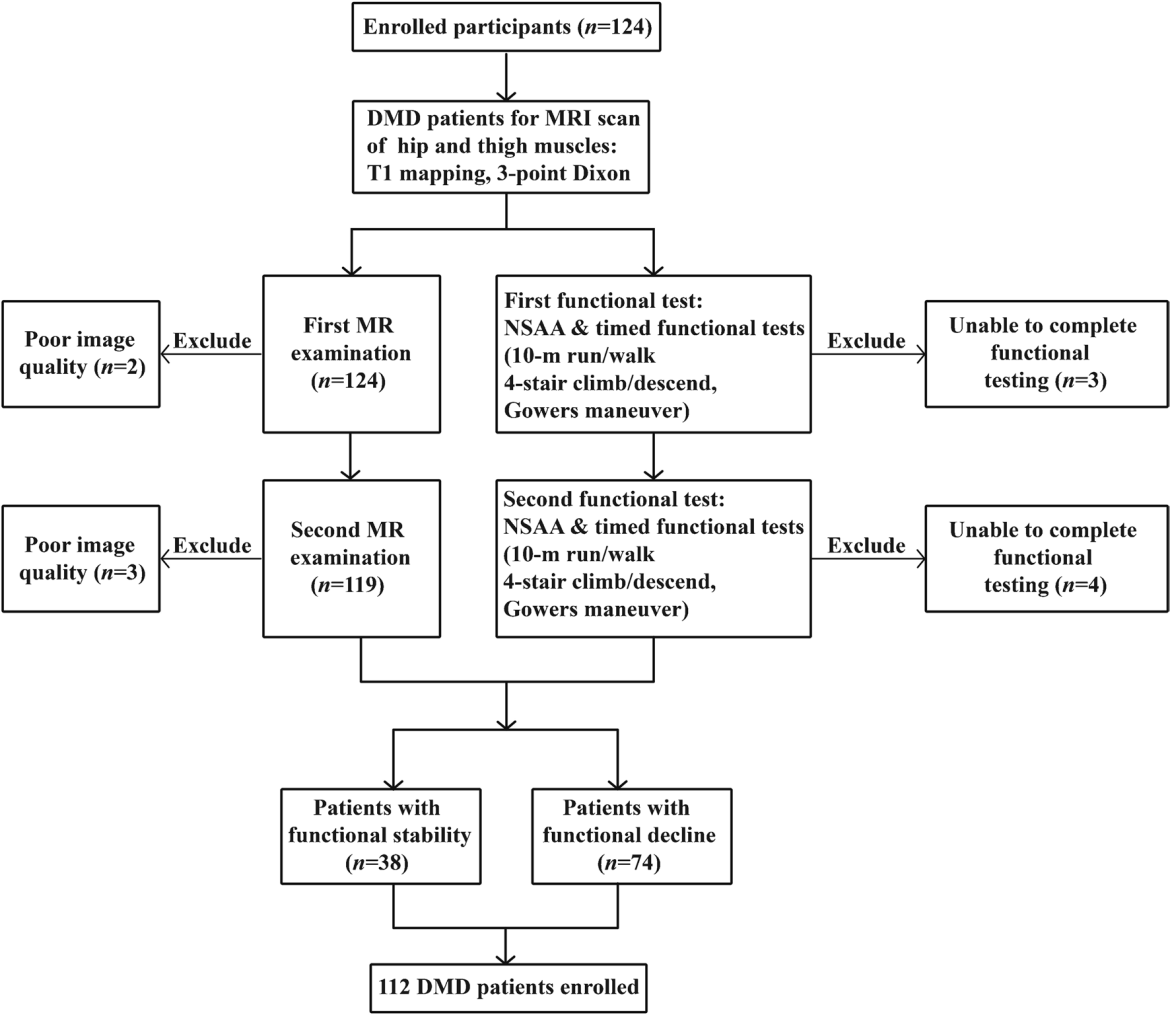
Study flow diagram describing recruitment of the patient cohort. *DMD* Duchenne muscular dystrophy, *MR(I)* magnetic resonance (imaging), *NSAA* North Star Ambulatory Assessment

Details of screening, exclusion, and eligibility for analysis of this study population are depicted in the study flow chart (Fig. 2). A total of 112 boys with DMD were enrolled. According to the changes in timed functional tests over 12 months, all participants were divided into two subgroups, including 38 subjects with functional stability and 74 subjects with functional decline (Table 1). Of the 112 subjects included, 98 had a history of taking corticosteroids at baseline, nine patients began corticosteroid therapy during the 12-month follow-up period, and the remaining five had not taken corticosteroids. At the 12-month follow-up time point, 112, 107, 104, 104, and 107 patients completed the North Star Ambulatory Assessment, 10m run/walk, 4-stair climb, 4-stair descend, and Gowers test, respectively.

**Table 1:** Participant characteristics and functional ability values

	Baseline (*n*=112)		12-month follow-up (*n*=112)	
	Stability (*n*=38)	Decline (*n*=74)	Stability (*n*=38)	Decline (*n*=74)
*Demographics*				
Age, y	8.6±2.0	9.4±1.5	9.7±1.9	10.5±1.5
Age, minimum/maximum, y	4.9/13.4	5.5/14.3	6.1/14.6	8.3/15.3
Height, cm	124.34±13.65	128.08±13.94	124.78±14.77	128.20±9.70
Weight, kg	26.82±8.18	28.26±7.15	29.62±9.28	31.91±8.29
BMI, kg/m^2^	17.13±2.91	17.90±2.58	17.83±3.30	19.16±2.89
Corticosteroid use, *n*^a^	28/10	70/4	33/5	74/0
Wheelchair use, *n*^a^	0/38	1/73	0/38	4/70
*Functional ability*				
NSAA score	73.00±17.92	47.46±16.60	75.81±17.48	38.41±18.14
10m run/walk, s	4.74±2.37	8.00±6.48	4.88±2.18	9.08±3.94
Gowers, s	4.16±4.86	10.28±11.67	4.55±4.69	16.16±16.82
4-stair climb, s	2.57±3.07	7.55±8.20	2.55±3.03	8.66±5.76
4-stair descend, s	2.04±1.63	5.12±5.72	1.87±1.30	6.19±5.90

Values are presented as the mean ± standard deviation

^a^For corticosteroid and wheelchair use, values represent on/off

*BMI* body mass index, *NSAA* North Star Ambulatory Assessment

### Longitudinal magnetic resonance biomarker trajectories

**Fig. 3:**
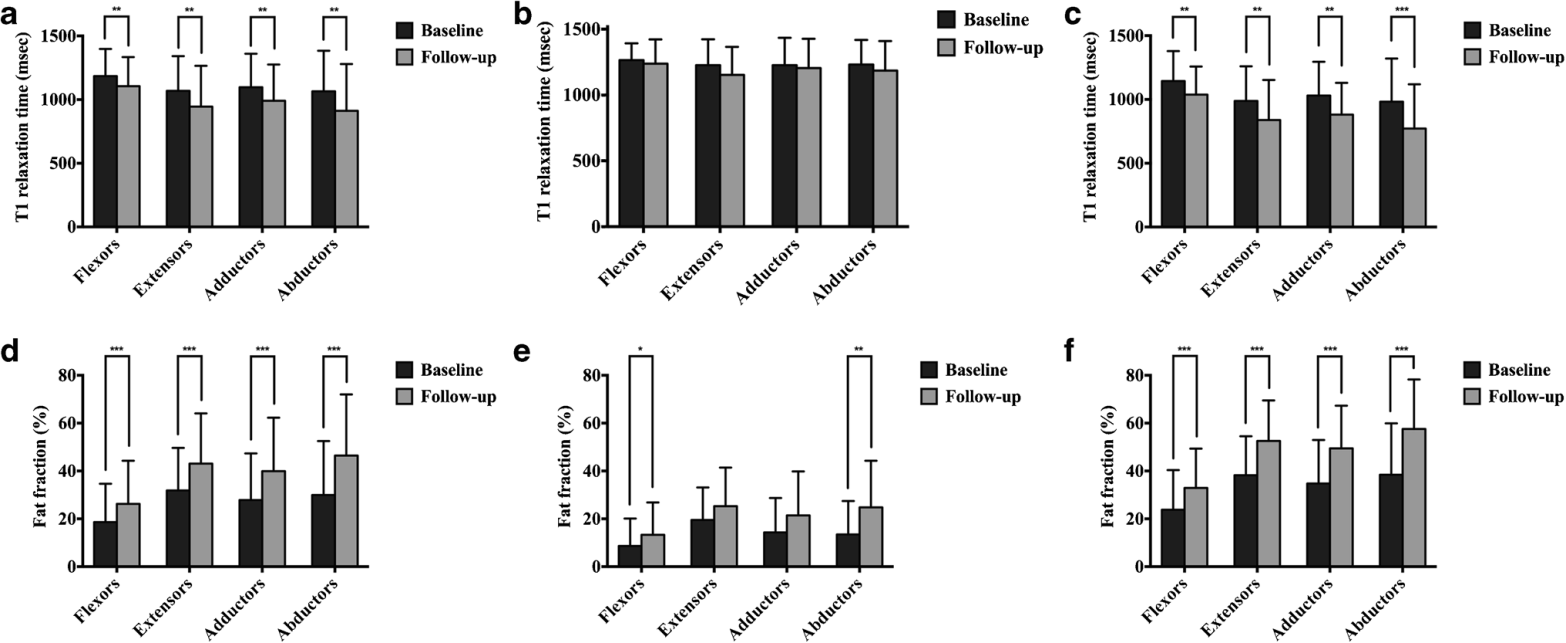
Longitudinal comparison of magnetic resonance biomarkers in gluteal muscle groups (T1 relaxation time, **a**–**c**; fat fraction, **d**–**f**) at baseline (*black bars*) and 12-month follow-up (*gray bars*). **a**, **d** All study subjects: all T1 values decreased and fat fraction values increased significantly over 12 months. **b**, **e** Functional stability group: only fat fraction of the flexors and abductors increased significantly over 12 months. **c**, **f** Functional decline group: all T1 values decreased and fat fraction values increased significantly over 12 months

Longitudinal comparisons of MR biomarkers at baseline and 12-month follow-up for all study subjects, the functional stability group and the functional decline group, are shown in Fig. 3. The longitudinal changes in the T1 and fat fraction values of the gluteal muscle groups over the 12-month period were different across subgroups. For all DMD patients, compared with baseline, the T1 of all gluteal muscle groups decreased significantly over 12 months, while fat fraction values increased significantly over 12 months (all *P*<0.05). For the functional stability group, compared with the baseline, there was no significant difference in T1 of all gluteal muscle groups and fat fraction of the extensors and adductors over 12 months (all *P*>0.05), while fat fraction of the flexors and abductors increased significantly over 12 months (all *P*<0.05). For the functional decline group, the T1 and fat fraction of all gluteal muscle groups were significantly different between the baseline and 12-month follow-up (*P*<0.05), with the T1 values being significantly lower than at baseline, while the fat fraction values were significantly higher than the baseline at 12-month follow-up. The T1 of the flexors, adductors, and abductors and the fat fraction of all gluteal muscle groups in the declining group were significantly higher than those in the stable group at 12 months (*P*<0.05) (Table 2). These results suggest that the changes in MR biomarkers in the functional decline group were more obvious than those in the functional stability group at the 12-month follow-up.

**Table 2:** Longitudinal changes in magnetic resonance biomarkers at baseline and at 12-month follow-up

	All subjects (*n*=112)		*P-*value	Functional stability group (*n*=38)		*P-*value	Functional decline group (*n*=74)		*P-*value	DMD 12-month follow-up		*P-*value
	Baseline	Follow-up		Baseline	Follow-up		Baseline	Follow-up		Stability	Decline	
*T1 (ms)*												
Flexors	1185.12±212.50	1105.96±227.99	0.005	1264.64±128.26	1238.08±183.95	0.339	1144.29±235.26	1038.12±219.40	0.005	21.77±175.94	−79.16±200.47	0.049
Extensors	1069.37±271.87	945.35±319.75	0.003	1226.48±196.32	1152.63±212.56	0.096	988.69±270.94	838.91±314.18	0.004	13.92±196.10	−124.01±250.85	0.097
Adductors	1096.81±263.40	991.06±284.38	0.005	1226.65±208.11	1204.94±222.19	0.575	1030.14±265.07	881.23±248.81	0.001	57.31±210.13	−105.75±197.08	0.001
Abductors	1065.35±318.47	912.33±366.95	0.001	1231.08±187.30	1185.58±224.34	0.299	982.48±338.59	772.02±347.00	0.000	83.81±373.80	−143.50±276.25	0.001
*Fat fraction (%)*												
Flexors	18.64%±16.00%	26.23%±18.06%	0.000	8.64%±11.47%	13.26%±13.54%	0.015	23.77%±15.62%	32.89%±16.45%	0.000	2.47%±4.38%	7.59%±7.84%	0.001
Extensors	31.86%±17.78%	43.07%±21.01%	0.000	19.50%±13.59%	25.22%±16.16%	0.082	38.20%±16.33%	52.54%±16.96%	0.000	4.43%±6.75%	11.22%±9.16%	0.000
Adductors	27.83%±19.51%	39.93%±22.37%	0.000	14.31%±14.37%	21.38%±18.37%	0.053	34.78%±18.16%	49.46%±17.84%	0.000	4.63%±6.08%	12.10%±8.60%	0.000
Abductors	29.93%±22.60%	46.44%±25.55%	0.000	13.44%±13.99%	24.73%±19.52%	0.002	38.40%±21.52%	57.58%±20.70%	0.000	8.15%±9.61¥	16.51%±13.27%	0.001

*DMD* Duchenne muscular dystrophy

### Relationship between baseline magnetic resonance biomarkers and 12-month follow-up functional ability

The relationship between baseline MR biomarkers and 12-month follow-up functional ability is presented in Table 3. The results show that all baseline T1 values were negatively correlated with age (r=−0.206 to −0.314, *P*<0.05), only baseline T1 of the extensors, adductors, and abductors were negatively correlated with height, weight, and BMI (r=−0.190 to −0.367, *P*<0.05), while all baseline fat fraction values were positively correlated with age, height, weight, and BMI (r=0.229 to 0.446, *P*<0.05). Overall, the baseline T1 value was positively correlated with the 12-month North Star Ambulatory Assessment score (r=0.480 to 0.620, *P*<0.001), but negatively correlated with the 12-month TFTs (Gowers, 10m run/walk, 4-stair climb, 4-stair descend) (r=−0.413 to −0.670, *P*<0.001). The baseline fat fraction value was negatively correlated with the 12-month North Star Ambulatory Assessment score (r=−0.701 to −0.783, *P*<0.001), but positively correlated with the 12-month timed functional tests (Gowers, 10 m run/walk, 4-stair climb, 4-stair descend) (r=0.596 to 0.787, *P*<0.001). Among all study subjects, baseline fat fraction of the flexors (r=−0.741, *P*<0.001), extensors (r=−0.701, *P*<0.001), adductors (r=−0.783, *P*<0.001) and abductors (r=−0.762, *P*<0.001) was strongly negatively correlated with the 12-month North Star Ambulatory Assessment, while baseline fat fraction of the adductors was strongly positively correlated with the 12-month Gowers (r=0.745, *P*<0.001), and baseline fat fraction of the flexors (r=0.736, *P*<0.001), adductors (r=0.787, *P*<0.001), and abductors (r=0.729, *P*<0.001) were strongly positively correlated with the 12-month 10m run/walk. Additionally, the baseline fat fraction of the adductors (r=0.737, *P*<0.001) and abductors (r=0.702, *P*<0.001) was strongly positively correlated with the 12-month 4-stair climb.

**Table 3:** Correlation between baseline magnetic resonance biomarkers and 12-month follow-up functional abilities in Duchenne muscular dystrophy

Variable	T1 (ms)				Fat fraction (%)			
	Flexors	Extensors	Adductor	Abductors	Flexors	Extensors	Adductor	Abductors
Age	−0.206^a^	−0.304^b^	−0.314^b^	−0.262^b^	0.360^b^	0.418^b^	0.422^b^	0.341^b^
Height	−0.167	−0.219^a^	−0.218^a^	−0.190^a^	0.297^b^	0.275^b^	0.317^b^	0.229^a^
Weight	−0.181	−0.350^b^	−0.307^b^	−0.313^b^	0.437^b^	0.446^b^	0.397^b^	0.417^b^
BMI	−0.178	−0.367^b^	−0.287^b^	−0.306^b^	0.396^b^	0.433^b^	0.317^b^	0.401^b^
Wheelchair use	−0.163	−0.137	−0.151	−0.158	0.163	0.157	0.163	0.160
NSAA	0.480^c^	0.510^c^	0.620^c^	0.554^c^	−0.741^c^	−0.701^c^	−0.783^c^	−0.762^c^
10m run/walk	−0.461^c^	−0.481^c^	−0.670^c^	−0.510^c^	0.736^c^	0.691^c^	0.787^c^	0.729^c^
Gowers	−0.455^c^	−0.463^c^	−0.625^c^	−0.422^c^	0.650^c^	0.623^c^	0.745^c^	0.650^c^
4-stair climb	−0.458^c^	−0.443^c^	−0.622^c^	−0.470^c^	0.686^c^	0.602^c^	0.737^c^	0.702^c^
4-stair descend	−0.444^c^	−0.413^c^	−0.557^c^	−0.483^c^	0.659^c^	0.596^c^	0.668^c^	0.655^c^

Significant differences are marked as ^a^*P*<0.05, ^b^*P*<0.01, ^c^*P*<0.0001

*BMI* body mass index, *NSAA* North Star Ambulatory Assessment

### Multivariable models for predicting future motor function decline

Table 4 shows the multivariable models. All baseline MRI biomarkers (T1 and fat fraction), age, and corticosteroid use were univariable factors associated with functional decline (all *P*<0.05). Variables with *P*<0.05 were included to construct the multivariate logistic regression model. Notably, the fat fraction of the flexors and adductors were put into two different logistic regression models **(**model 1 and model 2**)** due to the multicollinearity of these two covariates, while the other covariates were included in each of the two models. Specifically, model 1 has the variable “fat fraction of the flexors” but no variable “fat fraction of the adductors,” while model 2 has no variable “fat fraction of the flexors” but the variable “fat fraction of the adductors.” The T1 of the flexors, extensors, adductors, and abductors, fat fraction of the flexors, extensors, and abductors, age, and corticosteroid use were included to construct a multivariate logistic regression model **(**model 1**)**. The T1 of the flexors, extensors, adductors, and abductors, fat fraction of the extensors, adductors, and abductors, age, and corticosteroid use were included to construct another multivariate logistic regression model (model 2). The results showed that in both model 1 and model 2, baseline fat fraction of the abductors remained an independent risk factor for future motor function decline in DMD patients at 12 months (odds ratio [OR]=1.104, 95% confidence interval [CI]: 1.026~1.187, *P*=0.008; OR=1.085, 95% CI: 1.013~1.161, *P*=0.019).

**Table 4:** Univariate and multivariate logistic regression for predicting future motor function decline in Duchenne muscular dystrophy

Variable	Univariate analysis			Multivariate analysis (model 1)			Multivariate analysis (model 2)		
	OR	95% CI	*P*-value	OR	95% CI	*P*-value^a^	OR	95% CI	*P*-value^a^
*T1 (ms)*									
Flexors	0.997	0.994~0.999	0.006	1.000	0.995~1.005	0.914	1.001	0.996~1.005	0.832
Extensors	0.995	0.993~0.998	0.000	0.999	0.995~1.004	0.785	0.999	0.995~1.004	0.700
Adductors	0.997	0.995~0.998	0.001	1.002	0.998~1.006	0.444	1.002	0.998~1.007	0.292
Abductors	0.997	0.995~0.998	0.000	1.001	0.997~1.004	0.650	1.001	0.997~1.004	0.655
*Fat fraction (%)*									
Flexors	1.123	1.064~1.184	0.000	0.980	0.883~1.088	0.706	/	/	/
Extensors	1.098	1.055~1.143	0.000	1.026	0.940~1.121	0.564	1.003	0.920~1.094	0.941
Adductors	1.090	1.050~1.131	0.000	/	/	/	1.036	0.957~1.122	0.377
Abductors	1.099	1.055~1.145	0.000	1.104	1.026~1.187	**0.008**	1.085	1.013~1.161	**0.019**
Age(y)	1.338	1.033~1.733	0.028	1.071	0.764~1.499	0.691	1.038	0.735~1.467	0.831
BMI (kg/m^2^)	1.117	0.959~1.301	0.156	/	/	/	/	/	/
Corticosteroid use	6.250	1.809~21.589	0.004	3.915	0.621~24.687	0.146	3.857	0.663~22.435	0.133

*BMI* body mass index, *CI* confidence internal, *DMD* Duchenne muscular dystrophy, *OR* odds ratio

^a^*P*-value <0.05 is significant (bold)

**Fig. 4:**
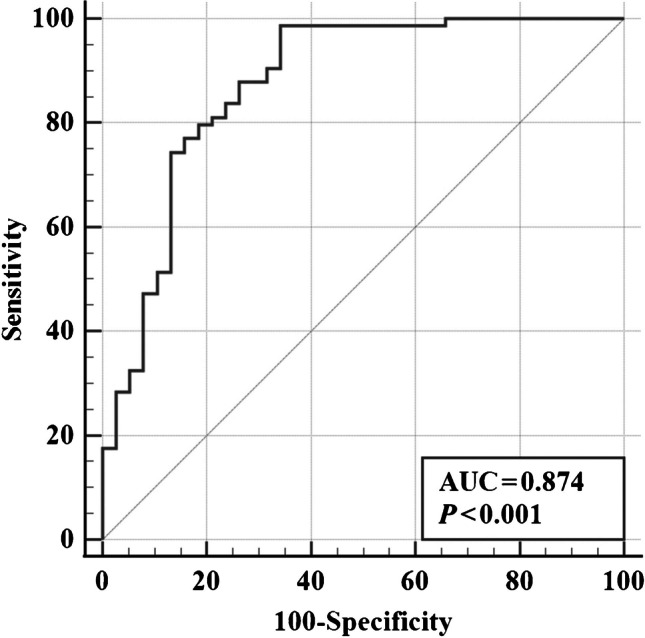
Receiver operating characteristic curve for the fat fraction of the abductors in predicting future motor function decline in Duchenne muscular dystrophy *AUC* area under the curve

ROC analysis (Fig. 4) was performed to investigate the diagnostic value of the baseline fat fraction of the abductors in predicting future motor function decline in DMD. ROC analysis showed that the baseline fat fraction of the abductors had a high predictive value for future motor function decline in DMD patients within 12 months, with an AUC of 0.874 and Youden’s index of 0.644. For fat fraction of the abductors, patients with a baseline >10.43% were more likely to experience a decline in motor function within 12 months, with a sensitivity of 98.7% and specificity of 65.8%.

## Discussion

This study demonstrates the value of MR biomarkers (T1 and fat fraction) in quantifying longitudinal changes in fat infiltration as a surrogate of DMD disease progression in gluteal muscle groups. The principal findings from this study are the following: (1) although the change in MR biomarkers (T1 and fat fraction) in the functional decline group was more obvious than that in the functional stability group over the following 12 months, fat fraction values in the functional stability group also increased during the 12-month follow-up, especially in flexors and abductors, suggesting that the pathology of these muscle groups is still progressing; (2) T1 value at baseline was positively correlated with North Star Ambulatory Assessment and negatively correlated with timed functional tests at the 12-month follow-up, while fat fraction value at baseline was negatively correlated with North Star Ambulatory Assessment and positively correlated with timed functional tests at the 12-month follow-up; and (3) fat fraction of the abductors has the potential to predict future clinical motor function decline within 12 months.

Many longitudinal studies have reported the progression of MR biomarkers in DMD individual muscles, mainly focusing on gluteal muscles, upper limb muscles, and lower limb muscles [23–26], but few studies have reported the longitudinal progression of MR biomarkers in gluteal muscle groups. In this study, we report the progression of MR biomarkers of gluteal muscle groups over 12 months and make some interesting findings. First, this study found that the longitudinal changes in the T1 and fat fraction values of the gluteal muscle groups during the 12-month follow-up varied according to patient subgroups: the T1 values of all gluteal muscle groups in the functional decline group significantly decreased, while the fat fraction values significantly increased. However, in the functional stability group, only the fat fraction of the flexors and abductors increased significantly. The progression of the fat fraction value in the functional stability group indicates that although patients in the functional stability group are still in the early stage of the disease with relatively moderate motor function, fat infiltration of the gluteal muscles is also occurring, which emphasizes that even in the early stage of DMD, only monitoring motor function is far from sufficient, and additional monitoring of the fat fraction value can provide more sensitive information and deserves attention in the clinic. The changes in MR biomarkers identified in this study are similar to the trend of the decrease in T1 value or the increase in fat fraction value of severely involved muscles in previous studies [15, 25, 27]. DMD is a progressive muscle degeneration disease [28], and pathological changes such as muscle injury, inflammation, and fat infiltration become increasingly severe, leading to a decrease in T1 [15] and an increase in fat fraction [17]. Second, we found that the changes in MR biomarkers in the functional decline group were more significant than those in the functional stability group over 12 months, which means the decrease in T1 and the increase in fat fraction values were more significant in the functional decline group. It is emphasized that the response of MR biomarkers is more sensitive in the functional decline group, namely, in the population of DMD patients with more severe disease. This may be attributed to the fact that patients in the functional stability group are still in the early stage of the disease, while patients in the functional decline group are in the middle or terminal stage of the disease. In the early stage of the disease, the T1 value is also affected by pathological changes such as fat infiltration, edema, and fibrosis. Edema increases T1, while fat decreases T1, and the two effects cancel each other out, resulting in the insignificant change in T1 values in the functionally stable group [29]. In the middle or terminal stage of the disease, fat infiltration dominates and the impact of fat on T1 is greater than that of edema, resulting in a decrease in T1 and an increase in fat fraction value [30]. Our results further emphasize the value of quantitative MR in assessing muscle injury in DMD patients under different physiological and pathological conditions and at different stages.

Although the correlation between MR biomarkers and functional ability in DMD has been demonstrated in previous cross-sectional studies [31, 32], the longitudinal relationship between them in the gluteal muscle group has not been fully expounded and explored. Our research shows that MR biomarkers of the gluteal muscle group are not only sensitive to longitudinal changes over time but also, more importantly, related to future functional ability. Among them, some baseline fat fraction values show a strong correlation with future functional ability, indicating that they are effective and sensitive early indicators of disease severity in DMD patients. Compared with the functional tests, acquisition of the fat fraction value requires less patient co-operation, and can be easily and quickly obtained even in patients with almost no walking ability in the middle or terminal stage of the disease. The correlation between baseline MR biomarkers and future clinical functional ability provides valuable information for quantitative MRI imaging to assess disease severity and/or to monitor treatment and supports the effectiveness of this noninvasive method to predict the progression of muscle involvement in DMD.

Finally, our data show that MR biomarkers can predict motor function decline over the following 12 months, which is of great significance for early clinical rehabilitation training and targeted therapy. Specifically, individuals with high fat fraction of the abductors (>10.5%) at baseline are likely to have decreased motor function in the following 12 months, while those with low fat fraction of the abductors at baseline (<10.5%) are likely to have stable motor function in the following 12 months. This suggests that MR biomarkers have the potential to provide early predictive cues before motor function decline occurs. Previous studies have reported that individuals with a high fat fraction value of the vastus lateralis may experience a decline in functional ability [11]. Combined with our data, these two studies illustrate the importance of evaluating the degree of muscle fat infiltration for predicting future motor function in DMD patients. However, our study focuses more on the involvement of gluteal muscle groups, with results suggesting that it is particularly important to pay attention to the changes in fat fraction of the abductors in the early stages of the disease, which can provide prognostic information on functional outcomes. The fat fraction of the abductors is highly sensitive to early muscle involvement, and these observations emphasize the necessity and feasibility of using related biomarkers to describe the natural history of diseases.

Several limitations are associated with this study. First, although the participants complied relatively well with the study, a few failed to complete each measurement at each study visit, especially in cases of more severe illness, resulting in loss of individual measurement data. However, most participants had complete data, so we believe these issues did not significantly affect our overall results. Second, because there were very few patients who completely lost ambulation in this study at the 12-month follow-up, we did not further explore the potential of MR biomarkers to predict future loss of ambulation, but this may be because our follow-up period of 12 months was not sufficiently long. We can further explore this in future longer-term follow-up studies. Third, due to the limited number of patients who did not receive steroid therapy, this study has not conducted a comparative longitudinal study between DMD boys who received steroid therapy and those who did not, and further studies are needed to determine the changes in MR biomarkers after steroid treatment in DMD patients.

## Conclusion

Fat fraction of the abductors is a powerful predictor of clinical motor function decline in DMD patients at 12 months, suggesting that quantitative MRI of the gluteal muscle groups may be used to noninvasively evaluate and predict disease progress.

## Data Availability

All datasets during this study are available from the corresponding author on reasonable request.
